# Calvaria Bone Transcriptome in Mouse Models of Osteogenesis Imperfecta

**DOI:** 10.3390/ijms22105290

**Published:** 2021-05-18

**Authors:** Pierre Moffatt, Iris Boraschi-Diaz, Juliana Marulanda, Ghalib Bardai, Frank Rauch

**Affiliations:** 1 Shriners Hospital for Children-Canada, Montreal, QC H4A 0A9, Canada; pmoffatt@shriners.mcgill.ca (P.M.); iris.boraschidiaz@mail.mcgill.ca (I.B.-D.); juliana.marulanda@mail.mcgill.ca (J.M.); GBardai@shriners.mcgill.ca (G.B.); 2 Department of Pediatrics, McGill University, Montreal, QC H4A 3J1, Canada; 3 Department of Human Genetics, McGill University, Montreal, QC H3A 0C7, Canada

**Keywords:** osteogenesis imperfecta, RNA sequencing, transforming growth factor beta, Wnt signaling

## Abstract

Osteogenesis imperfecta (OI) is a bone fragility disorder that is usually caused by mutations affecting collagen type I. We compared the calvaria bone tissue transcriptome of male 10-week-old heterozygous Jrt (*Col1a1* mutation) and homozygous *oim* mice (*Col1a2* mutation) to their respective littermate results. We found that Jrt and *oim* mice shared 185 differentially expressed genes (upregulated: 106 genes; downregulated: 79 genes). A total of seven genes were upregulated by a factor of two or more in both mouse models (*Cyp2e1*, *Slc13a5*, *Cgref1*, *Smpd3*, *Ifitm5*, *Cthrc1* and *Rerg*). One gene (*Gypa*, coding for a blood group antigen) was downregulated by a factor of two or more in both OI mouse models. Overrepresentation analyses revealed that genes involved in ‘ossification’ were significantly overrepresented among upregulated genes in both Jrt and *oim* mice, whereas hematopoietic genes were downregulated. Several genes involved in Wnt signaling and transforming growth factor beta signaling were upregulated in *oim* mice, but less so in Jrt mice. Thus, this study identified a set of genes that are dysregulated across various OI mouse models and are likely to play an important role in the pathophysiology of this disorder.

## 1. Introduction

Osteogenesis imperfecta (OI) is a heritable connective tissue disorder that is clinically characterized by low bone mass, bone fragility, long bone deformity, scoliosis, discolored sclera and dentinogenesis imperfecta [1]. In the large majority of individuals with OI, the condition is caused by mutations in *Col1a1* or *Col1a2* that directly affect the bone matrix protein collagen type I [2]. However, pathogenic variants in about 20 other genes can also give rise to an OI phenotype [1].

How mutations in *Col1a1* and *Col1a2* lead to low bone mass and bone fragility is not entirely clear. The low bone mass of OI is not simply caused by an inability of the osteoblast system to produce bone. Indeed, many children with severe OI have increased bone formation rates, as shown by iliac bone histomorphometry [3,4]. Low bone mass in OI is therefore likely caused by dysregulation of bone cell activity rather than the incapacity of osteoblasts to produce enough organic bone matrix.

Several mouse models with mutations in *Col1a1* or *Col1a2* are available to study the dysregulation of bone cells. A widely used model is the *oim* mouse that harbors a frameshift mutation in *Col1a2* [5]. Homozygous *oim* mice are small and have spontaneous fractures, mimicking the manifestations of severe OI in humans. The Jrt mouse is a model of dominant severe OI caused by a splice-site mutation in *Col1a1* that leads to an 18-amino acid deletion in the collagen type I alpha 1 chain [6]. Similar to the homozygous *oim* mouse, the heterozygous Jrt mouse has spontaneous fractures and bone deformities.

Thus, homozygous *oim* mice and heterozygous Jrt mice harbor quite different mutations in *Col1a2* and *Col1a1*, respectively, but share a similarly severe phenotype. Identifying molecular pathways and genes that are dysregulated in both mouse models might therefore help to delineate common factors that are involved in causing low bone mass and bone fragility in the context of mutations in collagen type I encoding genes. In the present study, we therefore performed RNA sequencing in the calvaria bone tissues of homozygous *oim* and heterozygous Jrt mice and performed differential gene expression analyses. We identified a set of genes that are dysregulated across various OI mouse models and are likely to play an important role in the pathophysiology of this disorder.

## 2. Results

**Figure 1:**
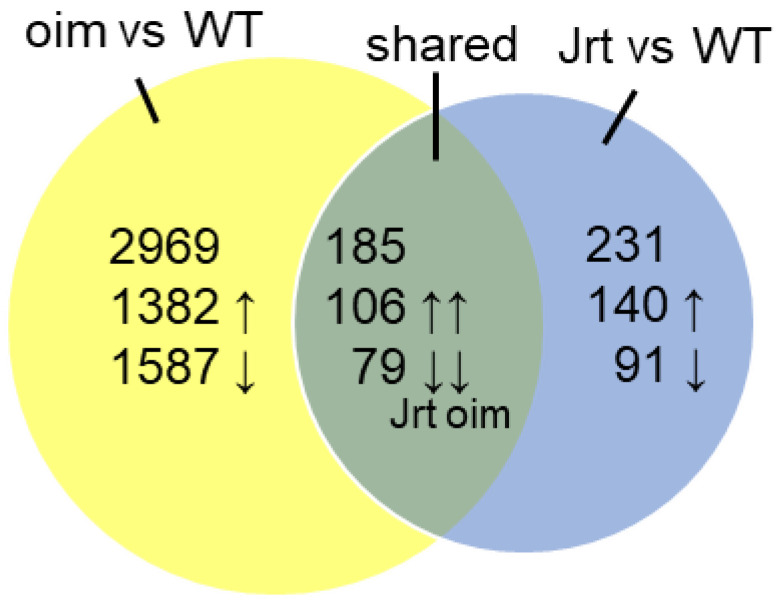
Results of RNA sequencing in calvaria of 10-week-old mice. Venn diagram of differentially expressed genes in *oim* and Jrt mice.

Compared to wild type (WT) mice, 231 genes were differentially expressed in Jrt mice, and 2969 genes were differentially expressed in *oim* mice (Figure 1). We found 185 genes that were differentially expressed in both Jrt and *oim* mice, which were all concordantly upregulated (106 genes) or downregulated (79 genes) between the two models (Supplementary Tables S1–S6).

The list of the top 20 upregulated genes in Jrt mice contained genes that are known to be important for the osteoblast function (e.g., *Bglap*—the gene coding for osteocalcin; *Ifitm5*—coding for BRIL) and the synthesis of extracellular matrix (*Smpd3*, *Col11a1*, *Col11a2* and *Adamts14*) (Table 1).

**Figure 2:**
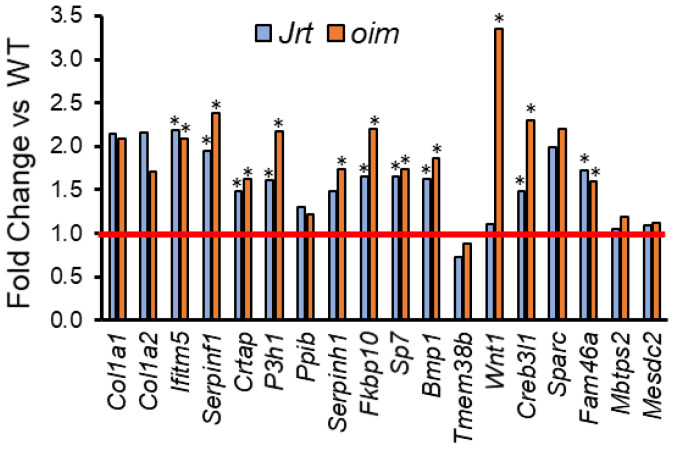
Fold change in Jrt and *oim* versus WT mice in genes that, when mutated, give rise to OI. The red line indicates parity to WT. Asterisks (*) indicate significant upregulated genes (adjusted *p* < 0.05, as determined by deseq2).

In *oim* mice, the list of the top 20 upregulated genes also contained genes that play a role in the function of osteoblasts (e.g., *Edn1*, *Ddit4*, *Cdkn1a*, *Igfbp3* and *Stc2*) (Table 2). Only one gene (*Cyp2e1*) was among the top 20 upregulated genes in both Jrt and *oim* mice. When all upregulated genes were considered, Jrt and *oim* mice shared several genes that play a role in the pathogenesis of various OI types (Figure 2).

**Figure 3:**
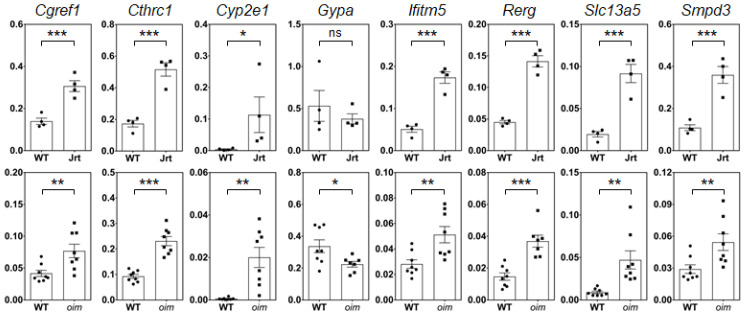
Real-time PCR results in calvaria RNA of 10-week-old male mice. Results for Jrt are shown in the upper panel, results for *oim* in the lower panel. Values represent the 2^−∆Ct^ normalized to *Rpl27*. Significant differences in gene expression compared to WT mice are indicated by asterisks (one-tailed unpaired *t*-test): ns, non-significant, * *p* < 0.05, ** *p* < 0.01, *** *p* < 0.001. Error bars represent standard errors.

To identify a core set of genes that are dysregulated in OI, we searched for genes where expression levels compared to WT mice differed by a factor of at least two in both OI models. A total of eight such genes were identified, seven of which were upregulated (*Cyp2e1*, *Slc13a5*, *Cgref1*, *Smpd3*, *Ifitm5*, *Cthrc1* and *Rerg*), and one (*Gypa*, coding for a blood group antigen) was downregulated (Table 3). The results for these eight genes were validated by RT-PCR (Figure 3). This confirmed the observations from the RNA sequencing analyses, even though the differences in *Gypa* levels were not significant in Jrt mice. As all previous experiments were conducted in male mice, we also verified differential gene expression in the calvaria of female *oim* mice using RT-PCR. Except for *Rerg* and *Smpd3*, results were similar to those of male *oim* mice (Supplementary Figure S1).

Six of the seven common upregulated genes (*Cyp2e1*, *Cgref1*, *Slc13a5*, *Cthrc1*, *Smpd3* and *Ifitm5*) were also upregulated in tibia osteocytes of *oim* mice, as previously reported by Zimmerman et al. [7]. In addition, four of these six genes (*Cgref1*, *Slc13a5*, *Smpd3* and *Ifitm5*) were upregulated in tibia osteocytes of CRTAP deficient mice, another mouse model of OI, as reported in the same study.

Overrepresentation analysis in Jrt mice showed that genes involved in ‘ossification’ (GO:0001503) were the most significantly enriched group among upregulated genes (enrichment ratio: 10.2) (Table 4). Other biological processes with significantly enriched upregulated genes included ‘extracellular structure organization’ (GO:0043062), ‘peptidyl-proline modification’ (GO:0018208) and ‘collagen metabolic process’ (GO:0032963). Genes involved in ‘myeloid cell differentiation’ (GO:0030099) were significantly overrepresented among downregulated genes (enrichment ratio: 8.2). Other biological processes significantly enriched for downregulated genes included ‘homeostasis of number of cells’ (GO:0048872) and ‘tetrapyrrole metabolic process’ (GO:0033013).

Overrepresentation analysis in *oim* mice revealed that genes involved in ‘ossification’ (GO:0001503) (enrichment ratio 4.0), ‘response to transforming growth factor beta’ (GO:0071559) (enrichment ratio 3.2) and ‘cell–cell signaling by Wnt’ (GO:0198738) (enrichment ratio 2.7) were significantly overrepresented among upregulated genes (false discovery rate <0.001 in each case) (Table 5). Among downregulated genes, genes involved in ‘myeloid cell differentiation’ (GO:0030099) (enrichment ratio: 2.6) were the most significantly overrepresented. The transcription factor target analysis revealed that there were no significantly enriched target gene sets in Jrt mice. In *oim* mice, there was a strong enrichment in genes that are responsive to SMAD3, SMAD4 (both of which are involved in TGF-beta signaling [8]) and FOXO1 (involved in Wnt signaling [9]) (false discovery rate <0.001 for each of these transcription factors) (Supplementary Table S7).

**Figure 4:**
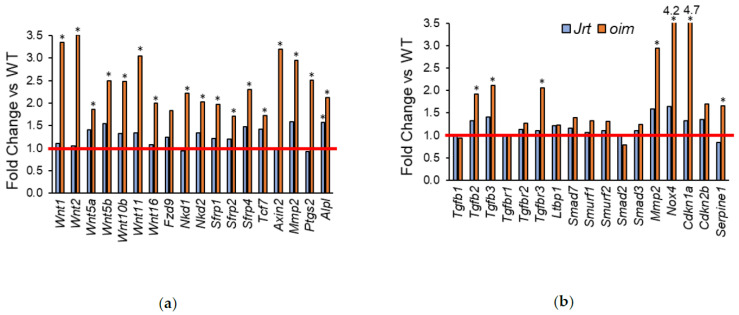
Fold change in Jrt and *oim* versus WT mice of genes that are involved in (**a**) Wnt signaling and (**b**) TGF-beta signaling. The red line indicates parity to WT. Asterisks (*) indicate significant upregulated genes (adjusted *p* < 0.05, as determined by deseq2).

In accordance with the biological processes identified by the overrepresentation analyses, several genes involved in Wnt signaling and TGF-beta signaling were upregulated in *oim* mice, but not, or to a lesser extent, in Jrt mice (Figure 4).

**Figure 5:**
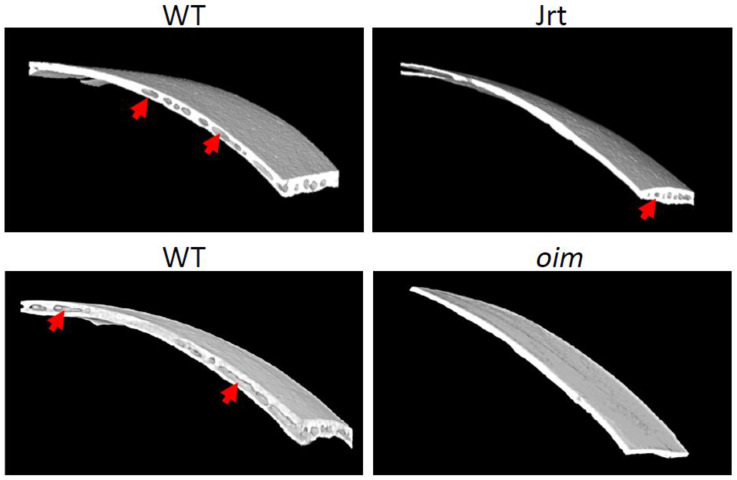
Three-dimensional rendering of microCT scans of 10-week-old WT, Jrt and *oim* mice’s right parietal bones. In Jrt and *oim* mice, the bone is thinner and contains less bone marrow space (arrows).

As the RNA sequencing analysis indicated that hematopoiesis was downregulated in OI mice, we hypothesized that this decrease in hematopoietic gene expression reflected a decrease in bone marrow space in the calvaria. We therefore performed microCT analysis of the calvaria bone of 10-week-old OI mice and their respective littermates. In Jrt and *oim* mice, the bone was thinner and contained less bone marrow space (Figure 5).

## 3. Discussion

In this study, we assessed differential gene expression in the calvaria bone tissues of two mouse models of severe OI, Jrt and *oim*. There were almost 13 times more differentially expressed genes in *oim* mice than in Jrt mice (2969 vs. 231 genes), and the list of the top 20 upregulated genes had little overlap between the two OI models. Genes involved in Wnt signaling and in TGF-beta signaling were upregulated in *oim* mice, but there was less evidence for dysregulation in these pathways in Jrt mice. Nevertheless, genes involved in ossification and genes that are involved in causing OI were upregulated in both OI models. We finally identified a set of genes that seem to be consistently upregulated across OI mouse models.

Why *oim* mice have many more upregulated genes than Jrt mice is not clear. Both are models of the severe OI that develop spontaneous fractures [5,6]. The *oim* model is homozygous for a *Col1a2* frameshift mutation, and therefore does not produce any normal collagen type I, whereas the Jrt mouse is heterozygous for a splice-site mutation that leads to the skipping of *Col1a1* exon 9 [5,6]. It is possible that the absence of normal collagen type I in the *oim* mouse triggers a larger number of regulatory cascades than the heterozygous abnormality in the Jrt mouse. It is also possible that the differences in transcriptome dysregulation are influenced by differences in the background strain, as *oim* mice in the present study were on a B6C3Fe background, whereas the Jrt mice were on an FVB background.

The observation that, compared to *oim*, Jrt mice seem to have milder or no abnormalities in Wnt and TGF-beta signaling pathways may explain our previous findings that Jrt mice are less responsive to treatments targeting these pathways [10,11]. We have previously noted that treatment with a sclerostin antibody, a therapeutic approach targeting Wnt signaling, appeared to be less effective in Jrt mice than in other mouse models of OI, including *oim* [10,12]. Similarly, the lower baseline activation of TGF-beta pathways in the Jrt mice may contribute to our observation that antibodies inactivating TGF-beta had less effect on bone mass in the Jrt mouse than in other OI models [11,13].

Among the strongly upregulated genes that are shared between Jrt and *oim* mice, *Cyp2e1*, *Slc13a5*, *Cgref1* and *Rerg* have not previously been implicated in OI pathophysiology to our knowledge. The fold change was highest for *Cyp2e1*. *Cyp2e1* codes for a cytochrome P450 enzyme that metabolizes many substrates and is involved in the generation of reactive oxygen species [14]. The functional importance of *Cyp2e1* in the bone is unclear, but prior studies have shown that *Cyp2e1* expression in osteoblasts decreases with unloading and increases with differentiation as well as after exposure to tumor necrosis factor alpha [15,16]. Systemically increased tumor necrosis alpha levels have been reported in *oim* mice [17]. In a separate study, we also observed increased *Cyp2e1* expression in the gastrocnemius muscle of both Jrt and *oim* mice, which supports the hypothesis that *Cyp2e1* is upregulated in these mice due to a systemic factor [18].

*Slc13a5* codes for a sodium-dependent citrate transporter that mediates the uptake of citrate from blood into many cell types, such as neurons, hepatocytes and osteoblasts [19]. Citrate plays not only a key role in cellular energy metabolism, but also in bone, as it binds to both apatite crystal surfaces and collagen in the extracellular matrix and thereby regulates crystal thickening [20]. Given that OI is characterized by abnormal crystal thickness [21], it seems plausible that citrate and Slc13a5 are involved in the abnormal mineralization seen in the OI bone. Interestingly, the *Slc13a5* deficient mouse has some phenotypic overlap with OI, as it shows amelogenesis imperfecta, a tooth enamel defect resembling dentinogenesis imperfecta as well as low bone mineral density [22]. Amelogenesis imperfecta is also present in humans with biallelic loss of function variants in *Slc13a5* [23]. It therefore appears plausible that the dysregulation of *Slc13a5* contributes to the clinical manifestations associated with OI.

*Cgref1* codes for a secretory protein that plays an important role in the regulation of the transcription factor AP-1 [24]. The role of *Cgref1* in osteoblasts or bones is not clear. *Rerg* encodes a transcriptional repressor that is much more highly expressed in calvarial osteoblasts than in femoral osteoblasts and may play a site-specific role in regulating estrogen signaling [25]. It is therefore interesting to note that *Rerg* was differentially regulated in male but not in female *oim* mice.

Several of the other strongly upregulated genes have a better characterized role in bones. *Ifitm5* codes for BRIL, a protein that is specifically found in osteoblasts [26]. A specific mutation in the 5′ untranslated region of *Ifitm5* gives rise to OI type V [27,28]. Although the exact role played by BRIL in regulating osteoblast activity has yet to be determined, *Ifitm5* has emerged as a specific and robust marker of osteoblast differentiation [29]. *Smpd3* encodes sphingomyelin phosphodiesterase 3, and *Smpd3* deficiency is associated with severe bone deformities, fractures and abnormal bone mineralization [30]. *Cthrc1* codes for a secreted osteoblast product that decreases bone resorption [31].

Regarding downregulated genes, those involved in ‘myeloid cell differentiation’ were overrepresented in both the Jrt and the *oim* model. *Gypa*, the one gene that was strongly downregulated in both Jrt and *oim* mice, codes for glycophorin A, which is one of the most abundant red cell proteins [32]. Our microCT analyses indicated that Jrt and *oim* mice had less bone marrow space in their calvaria bones. These observations suggest that the reduced expression of hematopoiesis genes in the OI calvaria reflects a decreased amount of hematopoietic bone marrow.

In conclusion, genes involved in Wnt signaling and in TGF-beta signaling were upregulated in *oim* mice, but there was less evidence for dysregulation in these pathways in Jrt mice. In both OI models, genes involved in ossification were upregulated, whereas hematopoietic genes were downregulated. This study also identified a set of genes that seem to be consistently upregulated in the bone tissues of several OI mouse models and therefore may play important roles in the pathophysiology of OI.

## 4. Materials and Methods

All experiments were approved by the Animal Care Committee of McGill University (protocol 2011-5975, approved on 1 April 2018) and conformed to the ethical guidelines of the Canadian Council on Animal Care. The Jrt mice on the FVB background were a gift from Dr. Jane Aubin’s laboratory, University of Toronto, Canada [6]. The breeding colony was maintained at the Animal Care Facility of the Shriners Hospitals for Children—Canada. Male heterozygous Jrt mice and their WT littermates were used for the experiments.

*Oim* mice on the B6C3Fe background were purchased from Jackson Laboratories (stock 4145670). Breeding pairs were mated to generate WT, heterozygote *oim*/+ and homozygote *oim* (hereafter designated *oim*). Only male WT and *oim* mice were used for the RNA sequence analyses, whereas real-time PCR tests were performed in both male and female mice. The colony was maintained by breeding *oim*/+ mice. Genotyping was performed by PCR as described by Zimmerman et al. [33].

### 4.1. Sample Preparation

Mice were euthanized at the age of 10 weeks (*n* = 4 for Jrt mice and the corresponding WT mice; *n* = 8 for *oim* and the corresponding WT). The calvariae were dissected from mice, immediately immersed in RNAlater (Ambion, Invitrogen, Austin, TX, USA) and stored at −20 °C until processing. While still in RNAlater, the calvariae were carefully cleaned of connective tissues. They were then cut along the sagittal and lambdoid sutures, such that only the parietal and frontal parts were used for the RNA extraction. After mincing with scissors, the bones were blotted to remove excess RNAlater and homogenized in 1 mL of TRIzol™ (Thermo Fisher Scientific, Waltham, MA, USA) for 15 s with a tissue mincer (OMNI International, Kennesaw, GA, USA) equipped with a 5-mm stainless steel generator probe. The total RNA was extracted and further purified on RNeasy MinElute columns (QIAGEN, Hilden, Germany) according to the manufacturer’s protocol.

### 4.2. Library Preparation and RNA Sequencing

Total RNA was quantified using a NanoDrop Spectrophotometer ND-1000 (NanoDrop Technologies, Inc., Wilmington, DE, USA), and its integrity was assessed by a 2100 Bioanalyzer (Agilent Technologies). The total RNA samples used in the present study had a concentration between 80 and 290 ng/μL, with an RNA integrity number ranging between 8.5 and 9.6. Libraries were generated from 250 ng of the total RNA as follows: mRNA enrichment was performed using the NEBNext Poly(A) Magnetic Isolation Module (New England Biolabs, Ipswich, MA, USA). cDNA synthesis was achieved with the NEBNext RNA First Strand Synthesis and NEBNext Ultra Directional RNA Second Strand Synthesis Modules (New England Biolabs). The remaining steps of the library preparation were done using the NEBNext Ultra II DNA Library Prep Kit for Illumina (New England Biolabs). Adapters and PCR primers were purchased from New England Biolabs. Libraries were quantified using the Quant-iT™ PicoGreen^®^ dsDNA Assay Kit (Life Technologies, Carlsbad, CA, USA) and the Kapa Illumina GA with Revised Primers—SYBR Fast Universal Kit (Kapa Biosystems, London, UK). Average size fragment was determined using a LabChip GX (PerkinElmer, Waltham, MA, USA) instrument.

The libraries were normalized, pooled and then denatured in 0.05 N NaOH and neutralized using HT1 buffer. The pool was loaded at 225 pM on an NovaSeq S4 l (Illumina, San Diego, CA, USA) ane using Xp protocol as per the manufacturer’s recommendations. The run was performed for 2 × 100 cycles (paired-end mode). A PhiX library was used as a control and mixed with libraries at 1% level. Base calling was performed with RTA v3. Program bcl2fastq2 v2.20 was then used to demultiplex samples and generate fastq reads. Between 36 million and 99 million 100-base paired-end reads were generated per sample.

#### Data Post-Processing and Statistical Evaluation

Analysis of paired-end sequencing reads was performed using GenPipes, open-source, Python-based standardized analysis pipelines, hosted on the Compute Canada High Performance Computing center (https://www.computecanada.ca). Briefly, paired-end sequencing reads were clipped for an adapter sequence, trimmed for a minimum quality (Q30) in 3’ and filtered for a minimum length of 32 bp using Trimmomatic [34]. Surviving read pairs were aligned to GRCm38 by the RNA-seq aligner STAR using the recommended two-passes approach [35]. Aligned RNA-seq reads were assembled into transcripts, and their relative abundance was estimated using Cufflinks [36] and Cuffdiff [37]. Exploratory analysis was conducted using various functions and packages from R and the Bioconductor project. Differential expression analysis was conducted using DESeq [38]. Expressed short single-nucleotide variants and indel calling were also performed by this pipeline. Significantly differentially expressed genes were defined as those with at least 5 fragments per kilobase of exon per million reads mapped level of expression and an adjusted *p* value < 0.05 as determined by DESeq. Biologically relevant patterns of differentially expressed genes were identified by Gene Set Enrichment Analysis using Reactome as implemented at the web-based GEne SeT AnaLysis Toolkit (WebGestalt) [39,40,41]. Transcription factor target analysis was also performed with WebGestalt.

### 4.3. Real-Time PCR Validation

Reverse transcription of 2 µg RNA was performed using the High Capacity cDNA Reverse Transcription Kit (Thermo Fisher, Waltham, MA, USA). Real-time PCR was performed in triplicate with 40 ng of cDNA using the QuantStudio™ 7 Flex System (Thermo Fisher), the TaqMan™ Fast Advanced Master Mix (Thermo Fisher) and the following FAM-labeled TaqMan^®^ Gene Expression primers: *Cgref1* (Mm01217831_m1), *Cthrc1* (Mm01163611_m1), *Cyp2e1* (Mm00491127_m1), *Gypa* (Mm00494848_m1), *Ifitm5* (Mm00804741_g1), *Rerg* (Mm02167721_m1), *Slc13a5* (Mm01334459_m1) and *Smpd3* (Mm00491359_m1). Results were normalized to *Rpl27* (Mm01245874_g1). Gene expression was analyzed according to the delta Ct method and expressed as 2^−∆Ct^.

### 4.4. Micro-Computed Tomography (MicroCT)

MicroCT of the calvaria was performed using a SkyScan 1272 device (Bruker, Billerica, MA, USA). The voxel size was 8 μm. Scan parameters included a 0.40-degree increment angle, 3 frames averaged, a 66 kV and 142 mA X-ray source with a 0.5-mm Al filter to reduce beam-hardening artifacts. Cortical analysis was performed in the right parietal bone, considering an ROI of 4-mm length and 2-mm width, between the sagittal and coronal suture. Scans were quantified using the system’s analysis software (SkyScan CT Analyzer, Version 1.16.1.0).

## Figures and Tables

**Table 1:** List of the top 20 upregulated genes in Jrt mice compared to WT mice. *p* values are adjusted by the Benjamini–Hochberg procedure, as determined by deseq2.

Gene	Description	Fold Change	*p*
*Cyp2e1*	Cytochrome P450, family 2, subfamily e, polypeptide 1	4.25	0.009
*Bglap*	Bone gamma carboxyglutamate protein	3.44	<0.001
*Bglap2*	Bone gamma-carboxyglutamate protein 2	3.14	<0.001
*Cyp2f2*	Cytochrome P450, family 2, subfamily f, polypeptide 2	2.90	<0.001
*Ighg2c*		2.87	0.02
*Slc13a5*	Solute carrier family 13, member 5	2.59	<0.001
*Creb3l3*	cAMP responsive element binding protein 3-like 3	2.55	0.003
*Tpsb2*	Tryptase beta 2	2.38	0.003
*Col11a1*	Collagen, type XI, alpha 1	2.34	0.006
*Col11a2*	Collagen, type XI, alpha 2	2.31	0.015
*Lipc*	Lipase, hepatic	2.30	<0.001
*Cgref1*	Cell growth regulator with EF hand domain 1	2.29	<0.001
*Ifi27l2a*	Interferon, alpha-inducible protein 27 like 2A	2.24	<0.001
*Smpd3*	Sphingomyelin phosphodiesterase 3, neutral	2.22	<0.001
*Ifitm5*	Interferon induced transmembrane protein 5	2.19	<0.001
*Ctsw*	Cathepsin W	2.14	0.04
*Kazald1*	Kazal-type serine peptidase inhibitor domain 1	2.14	<0.001
*Cthrc1*	Collagen triple helix repeat containing 1	2.05	<0.001
*Rerg*	RAS-like, estrogen-regulated, growth-inhibitor	2.05	0.002
*Adamts14*	A disintegrin-like and metallopeptidase with thrombospondin type 1 motif, 14	1.98	<0.001

**Table 2:** List of the top 20 upregulated genes in *oim* mice compared to WT mice. *p* values are adjusted by the Benjamini–Hochberg procedure, as determined by deseq2.

Gene	Description	Fold Change	*p*
*Mt2*	Metallothionein 2	7.32	0.004
*Edn1*	Endothelin 1	7.10	<0.001
*Aspg*	Asparaginase	6.37	0.002
*Slc10a6*	Solute carrier family 10, member 6	5.71	<0.001
*Npas2*	Neuronal PAS domain protein 2	5.51	<0.001
*Cyp2e1*	Cytochrome P450, family 2, subfamily e, polypeptide 1	5.51	<0.001
*Angptl7*	Angiopoietin-like 7	5.27	0.005
*Ddit4*	DNA-damage-inducible transcript 4	4.99	<0.001
*Arl4d*	ADP-ribosylation factor-like 4D	4.74	<0.001
*Cdkn1a*	NUS1 dehydrodolichyl diphosphate synthase subunit	4.74	<0.001
*Sult5a1*	Sulfotransferase family 5A, member 1	4.71	<0.001
*Cxcl13*	Chemokine (C-X-C motif) ligand 13	4.69	<0.001
*Zbtb16*	Zinc finger and BTB domain containing 16	4.67	<0.001
*Adamts15*	A disintegrin-like and metallopeptidase with thrombospondin type 1 motif, 15	4.66	<0.001
*Igfbp3*	Insulin-like growth factor binding protein 3	4.35	<0.001
*Mt1*	Metallothionein 1	4.35	<0.001
*Stc2*	Stanniocalcin 2	4.35	<0.001
*Syt13*	Synaptotagmin XIII	4.33	<0.001
*Itga10*	Integrin, alpha 10	4.32	<0.001
*Adm*	Adrenomedullin	4.31	0.001

**Table 3:** Genes that are up- or downregulated 2-fold or more in the calvaria of both Jrt and *oim* mice. *p* values are adjusted by the Benjamini–Hochberg procedure, as determined by deseq2.

Gene	Description	Jrt		*oim*	
		Fold Change	*p*	Fold Change	*p*
Upregulated					
*Cyp2e1*	Cytochrome P450, family 2, subfamily e, polypeptide 1	4.25	0.009	5.51	<0.001
*Slc13a5*	Solute carrier family 13, member 5	2.29	<0.001	3.30	<0.001
*Cgref1*	Cell growth regulator with EF hand domain 1	2.05	<0.001	2.87	<0.001
*Smpd3*	Sphingomyelin phosphodiesterase 3, neutral	2.59	<0.001	2.70	0.02
*Ifitm5*	Interferon induced transmembrane protein 5	2.05	<0.001	2.54	<0.001
*Cthrc1*	Collagen triple helix repeat containing 1	2.22	<0.001	2.51	<0.001
*Rerg*	RAS-like, estrogen-regulated, growth-inhibitor	2.19	0.002	2.09	<0.001
Downregulated					
*Gypa*	Glycophorin A	0.45	<0.001	0.44	0.01

**Table 4:** Overrepresentation analysis of genes that are differentially expressed in Jrt mice. FDR: false discovery rate.

Gene Set	Description	FDR	Genes in Set
Upregulated			
GO:0001503	ossification	<0.001	*Alpl*; *Aspn*; *Bglap*; *Bglap2*; *Bmp1*; *Bmp3*; *Bmp8a*; *Col11a1*; *Col11a2*; *Creb3l1*; *Cthrc1*; *Dkk1*; *Dmp1*; *Gdf10*; *Gja1*; *Ifitm5*; *Igsf10*; *Kazald1*; *Lrrc17*; *Mepe*; *Omd*; *Ostn*; *P3h1*; *Phex*; *Phospho1*; *Pth1r*; *Smad6*; *Sp7*; *Tmem119*; *Twist1*
Downregulated			
GO:0030099	myeloid cell differentiation	<0.001	*Alas2*; *Ank1*; *Bpgm*; *Car2*; *Dmtn*; *Epb42*; *Hmgb2*; *Myb*; *Rhag*; *Rhd*; *Spib*; *Tal1*; *Trim10*; *Trim58*; *Zfpm1*

**Table 5:** Overrepresentation analysis of genes that are differentially expressed in *oim* mice. FDR: False discovery rate.

Gene Set	FDR	Genes in Set
Upregulated		
GO:0001503 (ossification)	<0.001	*Acvr1*; *Alpl*; *Ank*; *Ano6*; *Atraid*; *Axin2*; *Bcl2*; *Bmp1*; *Bmp2*; *Bmp3*; *Bmp4*; *Bmp8a*; *Bmpr1a*; *Bmpr2*; *Cd276*; *Cebpb*; *Cebpd*; *Chrdl1*; *Clec11a*; *Col11a1*; *Col11a2*; *Col13a1*; *Col5a2*; *Creb3l1*; *Cthrc1*; *Dchs1*; *Ddr2*; *Dlx5*; *Dmp1*; *Ecm1*; *Epha2*; *Ext1*; *Ext2*; *Fam20c*; *Fat4*; *Fgfr1*; *Fgfr2*; *Fndc3b*; *Foxc1*; *Foxc2*; *Fzd1*; *Gabbr1*; *Gja1*; *Gli1*; *Gpc3*; *Gpm6b*; *Gpnmb*; *Hspg2*; *Ibsp*; *Id3*; *Id4*; *Ifitm5*; *Igf2*; *Igfbp3*; *Igsf10*; *Il6st*; *Intu*; *Jag1*; *Kazald1*; *Kremen1*; *Lgr4*; *Lrp4*; *Lrp5*; *Lrp6*; *Lrrc17*; *Ltbp3*; *Mepe*; *Mgp*; *Mia3*; *Mmp14*; *Mmp2*; *Mn1*; *Nab2*; *Nog*; *Npnt*; *Npr2*; *Omd*; *P3h1*; *Phex*; *Pkdcc*; *Ptch1*; *Pth1r*; *Ptk2*; *S1pr1*; *Sfrp1*; *Sfrp2*; *Sh3pxd2b*; *Sik3*; *Six2*; *Smad1*; *Smad6*; *Smo*; *Smoc1*; *Snai1*; *Sost*; *Sp7*; *Tgfb2*; *Tgfbr3*; *Thbs3*; *Thra*; *Tmem119*; *Tnfsf11*; *Tnn*; *Trpm4*; *Twist1*; *Twsg1*; *Wnt10b*; *Wwtr1*; *Zbtb16*; *Zhx3*
Downregulated		
GO:0030099 (myeloid cell differentiation)	<0.001	*B2m*; *Adar*; *Fes*; *Rcor1*; *G6pdx*; *Nrros*; *Itgb3*; *Casp8*; *Tyrobp*; *Ubd*; *Creb1*; *Pde1b*; *Tmem14c*; *Fcer1g*; *Pf4*; *Fli1*; *Wdr1*; *Tcf3*; *Senp1*; *Pml*; *Atpif1*; *Plscr1*; *Myh9*; *Pknox1*; *Ubash3b*; *Rb1*; *Spi1*; *Casp3*; *Kit*; *Adam8*; *Nckap1l*; *Smarca4*; *Ets1*; *Tspan2*; *Eif2ak1*; *Hcls1*; *Isg15*; *Ptbp3*; *Pabpc4*; *Inpp5d*; *Cd300lf*; *Lyn*; *Clec2i*; *Ptk2b*; *Ankle1*; *Mpl*; *Ltf*; *Ncapg2*; *Hmgb3*; *Clec1b*; *Ptpn6*; *Pilrb1*; *Cdk6*; *Irf8*; *Clec5a*; *Prtn3*; *Tfrc*; *Tesc*; *Ceacam1*; *Cd101*; *Lmo2*; *Irf4*; *Csf3r*; *Hhex*; *Stap1*; *Cebpe*; *Bpgm*; *Hmgb2*; *Ank1*; *Gfi1b*; *Gata1*; *Spib*; *Dmtn*; *Zfpm1*; *Rhd*; *Alas2*; *Rhag*; *Tal1*; *Dyrk3*; *Car2*; *Myb*; *Epb42*; *Trim10*; *Trim58*

## Supplementary Materials

The following are available online at https://www.mdpi.com/article/10.3390/ijms22105290/s1, Figure S1: Real-time PCR results in calvaria RNA of 10-week-old female mice, Table S1: Genes upregulated in Jrt mice, Table S2: Genes downregulated in Jrt mice, Table S3: Genes upregulated in *oim* mice, Table S4: Genes downregulated in *oim* mice, Table S5: Genes upregulated in both Jrt and *oim* mice, Table S6: Genes downregulated in both Jrt and *oim* mice, Table S7. Results of transcription factor target analysis in male oim mice.
