# Supplementary material for: Calvaria Bone Transcriptome in Mouse Models of Osteogenesis Imperfecta
Source: Int J Mol Sci. 2021 May 18;22(10):5290. doi: 10.3390/ijms22105290 (PMC8157281; doi:10.3390/ijms22105290)
Supplement: Supplementary file 1 [file ijms-22-05290-s001.zip › Supplemental Figure1.pdf]

Supplemental Figure 1

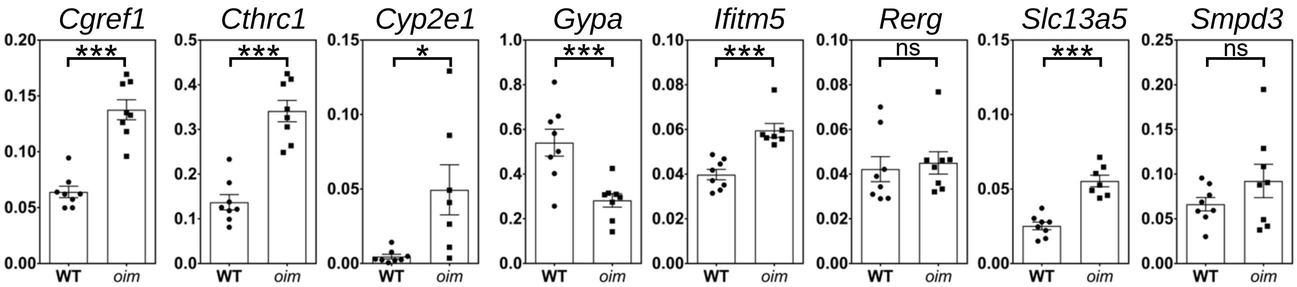

Supplemental Figure 1. Real-time PCR results in calvaria RNA of 10-week-old female *oim* mice. Values represent the $2^{-\Delta Ct}$ normalized to *Rpl27*. Significant differences in gene expression compared to WT mice are indicated by asterisks (one-tailed unpaired t test): ns, non-significant, * p<0.05, *** p < 0.001. Error bars represent standard errors.
